# Case Report: ANXA2 Associated Life-Threatening Coagulopathy With Hyperfibrinolysis in a Patient With Non-APL Acute Myeloid Leukemia

**DOI:** 10.3389/fonc.2021.666014

**Published:** 2021-04-15

**Authors:** Leo Ruhnke, Friedrich Stölzel, Lisa Wagenführ, Heidi Altmann, Uwe Platzbecker, Sylvia Herold, Andreas Rump, Evelin Schröck, Martin Bornhäuser, Johannes Schetelig, Malte von Bonin

**Affiliations:** ^1^ Department of Internal Medicine I, University Hospital Carl Gustav Carus, TU Dresden, Dresden, Germany; ^2^ National Center for Tumor Diseases (NCT) Dresden, Dresden, Germany; ^3^ German Cancer Consortium (DKTK) partner site Dresden, Dresden, Germany; ^4^ German Cancer Research Center (DKFZ), Heidelberg, Germany; ^5^ Medical Clinic and Policlinic I, Hematology and Cellular Therapy, Leipzig University Hospital, Leipzig, Germany; ^6^ Institute of Pathology, University Hospital Carl Gustav Carus, TU Dresden, Dresden, Germany; ^7^ Institute of Clinical Genetics, Carl Gustav Carus Faculty of Medicine, TU Dresden, Dresden, Germany

**Keywords:** acute promyelocytic leukemia, acute myeloid leukemia, hyperfibrinolysis, disseminated intravascular coagulation, coagulopathy, ANXA2, PDPN, WES

## Abstract

Patients with acute promyelocytic leukemia (APL) often present with potentially life-threatening hemorrhagic diathesis. The underlying pathomechanisms of APL-associated coagulopathy are complex. However, two pathways considered to be APL-specific had been identified: 1) annexin A2 (ANXA2)-associated hyperfibrinolysis and 2) podoplanin (PDPN)-mediated platelet activation and aggregation. In contrast, since disseminated intravascular coagulation (DIC) is far less frequent in patients with non-APL acute myeloid leukemia (AML), the pathophysiology of AML-associated hemorrhagic disorders is not well understood. Furthermore, the potential threat of coagulopathy in non-APL AML patients may be underestimated. Herein, we report a patient with non-APL AML presenting with severe coagulopathy with hyperfibrinolysis. Since his clinical course resembled a prototypical APL-associated hemorrhagic disorder, we hypothesized pathophysiological similarities. Performing multiparametric flow cytometry (MFC) and immunofluorescence imaging (IF) studies, we found the patient’s bone-marrow mononuclear cells (BM-MNC) to express ANXA2 - a biomarker previously thought to be APL-specific. In addition, whole-exome sequencing (WES) on sorted BM-MNC (leukemia-associated immunophenotype (LAIP)1: ANXA^lo^, LAIP2: ANXA^hi^) demonstrated high intra-tumor heterogeneity. Since ANXA2 regulation is not well understood, further research to determine the coagulopathy-initiating events in AML and APL is indicated. Moreover, ANXA2 and PDPN MFC assessment as a tool to determine the risk of life-threatening DIC in AML and APL patients should be evaluated.

## Introduction

Patients with APL often present with potentially life-threatening hemorrhagic diathesis (1). Here, pathophysiology of APL-associated coagulopathy is complex (2). Beside enhanced thrombin activation inducing disseminated intravascular coagulation (DIC), annexin A2 (ANXA2) mediated hyperfibrinolysis has been identified as a key-pathway (3). ANXA2, encoded by the *ANXA2* gene located on chromosome 15, serves as a cell surface receptor for both, plasminogen (PLG) and tissue-type plasminogen activator (PLAT), accelerating plasmin formation (4). Thus, ANXA2 overexpression on APL promyelocytes is thought to cause hyperfibrinolysis (5).

In addition to the well-known mediators of APL-associated coagulopathy, such as tissue factor, ANXA2 and PLAT, podoplanin (PDPN) - previously known as a marker of lymphatic endothelial cells - has been recently identified as a contributing factor to APL-associated hemorrhagic disorders (6). PDPN, expressed on APL cells, leads to platelet activation and aggregation, thus causing thrombocytopenia, prolonged bleeding time as well as venous and arterial thrombosis (7).

In contrast, coagulopathy/hyperfibrinolysis is less frequent in patients with non-APL AML. Therefore, 1) the potential threat of coagulopathy in non-APL AML patients may be underestimated and 2) underlying pathophysiology of AML-associated hemorrhagic disorders is not well understood.

## Case Description

A 64-year-old man (unique patient number (UPN) 1) noticed spontaneous bruising of the arms and legs about six weeks before admission, at the same time progressive fatigue and night sweats developed. Due to episodes of spontaneous, prolonged gingival hemorrhage the patient presented to his primary care doctor two weeks later, whereupon the patient was referred to our center. On admission, the patient´s white blood cell (WBC) count was 5.3 x 10^9^/L (normal: 3.8-9.8 x 10^9^/L), hemoglobin was 4.65 mmol/L (normal: 8,6-12,1 mmol/L), and platelet count was 51,000 x 10^9^/L (normal 150,000-400,000 x 10^9^/L) with a WBC differential including 27% neutrophils, 25% monocytes, 38% lymphocytes, 1% eosinophils, and 9% myeloblasts. Prothrombin ratio (PR) was 49% (normal: 70-120%), activated partial thromboplastin time (aPTT) 40 s (normal: 24-36 s), initial fibrinogen level 1,28 g/L (normal: 2-4 g/L), D-dimer level >4 µg/mL FEU (normal: <0,5 µg/mL FEU), antithrombin activity 112% (normal: 80-120%), and the International Society on Thrombosis and Haemostasis (ISTH) DIC score was 6 (normal < 5) all together indicating overt DIC with ongoing hyperfibrinolysis (8). A bone marrow biopsy was performed and the diagnosis of AML with maturation (FAB M2) was established. Further workup revealed a normal male karyotype, a partial tandem duplication of *KMT2A* (*KMT2A*-PTD) as well as an *IDH2* and a *SRSF2* mutation (no *CBFB-MYH11*, *PML-RARA* or *RUNX1-RUNX1T1* fusion, no *ASXL1*, *CEBPA*, *FLT3*, *NPM1, RUNX1 or TP53* mutation).

**Figure 1:**
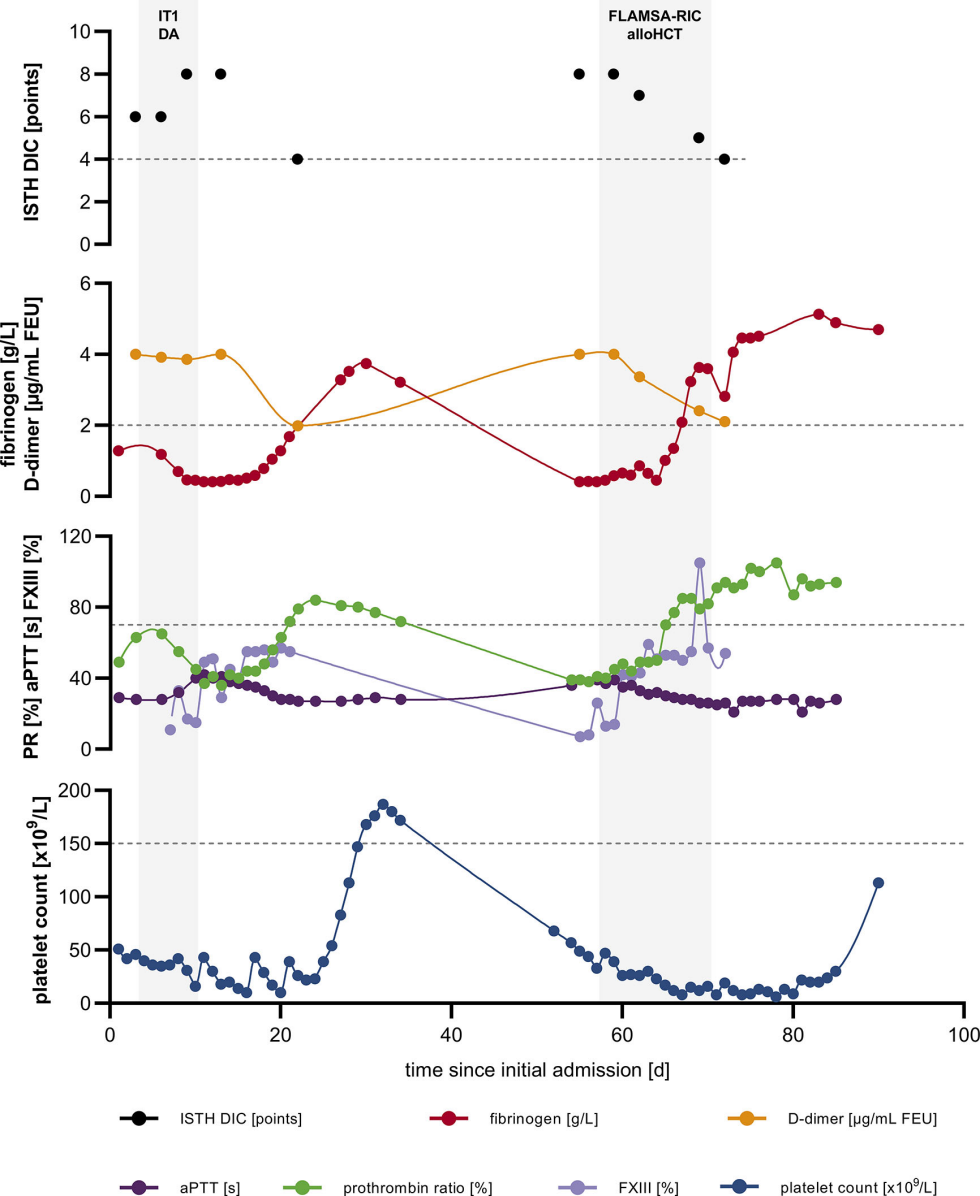
Severe, recurring coagulopathy with hyperfibrinolysis. ISTH DIC score; fibrinogen and D-dimer levels; PR, aPTT and FXIII as well as platelet count over time; dashed lines indicate upper limit of normal (ULN) for ISTH DIC score and lower limit of normal (LLN) for fibrinogen, PR, platelet count, respectively. alloHCT, allogeneic hematopoietic cell transplantation; aPTT, activated partial thromboplastin time; DIC, disseminated intravascular coagulation; FLAMSA-RIC, sequential conditioning regimen (fludarabine, amsacrine and cytarabine followed by fludarabine, busulfan and anti-thymocyte globulin); FXIII, factor XIII activity; IT1 DA, induction chemotherapy: daunorubicin and cytarabine; ISTH, International Society on Thrombosis and Haemostasis; PR, prothrombin ratio.

Following initiation of induction chemotherapy with a standard “7+3” regimen, hemorrhagic diathesis worsened: severe hyperfibrinolysis with diffuse mucosal and post-interventional bleeding occurred. Beside extensive transfusion of platelets, fibrinogen, prothrombin complex concentrate (PCC), and factor XIII, application of tranexamic acid was required to counteract coagulopathy and stabilize fibrinogen levels. About two weeks following admission hemorrhagic diathesis began to resolve and we could deescalate the transfusion regimen (**Figure 1**).

Due to 1) primary refractory disease and 2) the adverse prognosis associated with *KMT2A-*PTD, the patient was assigned to undergo matched-unrelated hematopoietic cell transplantation (HCT) (9).

Owing to recurrent gingival and hemorrhoidal bleeding the patient was admitted to the HCT unit earlier than scheduled. On re-admission, the patient´s white blood cell (WBC) count was 1.93 x 10^9^/L (normal: 3.8-9.8 x 10^9^/L), hemoglobin was 4.9 mmol/L (normal: 8,6-12,1 mmol/L), and platelet count was 57,000 x 10^9^/L (normal 150,000-400,000 x 10^9^/L) with a WBC differential including 9% neutrophils, 31% monocytes, 49% lymphocytes, 2% eosinophils, 1% basophils and 8% myeloblasts. Fibrinogen level was <0,4 g/L (normal: 2-4 g/L), D-dimer level >4 µg/mL FEU (normal: <0,5 µg/mL FEU), PR 39% (normal: 70-120%), aPTT 36 s (normal: 24-36 s), antithrombin activity 99% (normal: 80-120%), and ISTH DIC score was 8 (normal: <5), resembling the situation at initial presentation (**Figure 1**). Once more extensive supplementation of platelets, fibrinogen and tranexamic acid was required to control ongoing coagulopathy. Simultaneously, a sequential conditioning regimen according FLAMSA-RIC (fludarabine, amsacrine and cytarabine followed by fludarabine, busulfan and anti-thymocyte globulin) was performed since the patient did not achieve complete remission. On day 70 following initial presentation the patient underwent peripheral blood HCT from a matched unrelated donor with HLA-DPB1 non-permissive mismatch. Cyclosporine A and mycophenolate mofetil were administered as graft-versus-host disease (GvHD) prophylaxis. Beyond grade 3 mucositis and neutropenic fever, further aplasia took a complication-free course and platelet and neutrophil engraftment occurred on day +14 and +18 following HCT, respectively.

Two months after transplantation, examination of a bone marrow–biopsy specimen revealed complete morphologic and molecular remission and a full donor chimerism. On last follow-up, 12 months after transplantation, the patient is free of complaints and remains in complete remission.

## Methods

We determined ANXA2 and PLAT expression on the patient´s (UPN1) bone marrow derived mononucleated cells (BM-MNCs) *via* multiparametric flow cytometry (MFC) and immunofluorescence staining of cytospin samples (IF). Furthermore, PDPN expression was examined *via* IF. Further, whole exome sequencing (WES) was performed on sorted UPN1 BM-MNCs populations: Briefly, DNA - isolated from BM-MNC populations - was quantified using the Qubit dsDNA HS Assay (Q32851, Life Technologies). Library construction was performed from isolated DNA using TruSeq DNA Nano Sample Preparation kits (Illumina, San Diego, California, USA) according to the manufacturer’s instructions and indexed libraries were paired end (2x151 bp) sequenced on Illumina HiSeqX instrument (Illumina). FASTQ files were generated using the BCL2 fastQ pipeline (bcl2fastq 2.19.0.316). Per sample, on average 498M (range 455M-514M) read ends were obtained. HG19 was used as reference genome for bioinformatic analyses. The bioinformatics evaluation was performed using the Biomedical Workbench from CLC (12.0.3) using a customized analysis algorithm with following filters: coverage >/=25, variant allele frequency >/=10%. SNVs were further annotated for their biological effect and filtered based on SNV-quality, minimum number of supporting reads and biological relevance (non-synonymous SNVs). Variant assessment/classification was performed using VarSome, which includes—among others—ClinVar and dbSNP databases, population frequency information from gnomAD and in-silico prediction tools such as FATHMM, SIFT, REVEL and Polyphen2 (10). Only variants classified as pathogenic, likely pathogenic or variants of uncertain significance (VUS) - according to the American College of Medical Genetics and Genomics (ACMG) - were reported (11). BM-MNCs obtained from a patient with APL (UPN 2), a patient diagnosed with AML (FAB M2) with *KMT2A-*PTD and an *IDH2* mutation without evidence of DIC (UPN3), and a healthy donor served as positive and negative control, respectively. Here, BM-MNCs were obtained from the Study Alliance Leukemia (SAL) biobank, which has been approved by the Ethics Committee of the TU Dresden (EK98032010). Written informed consent had been obtained from all participants.

## Results and Discussion

**Figure 2:**
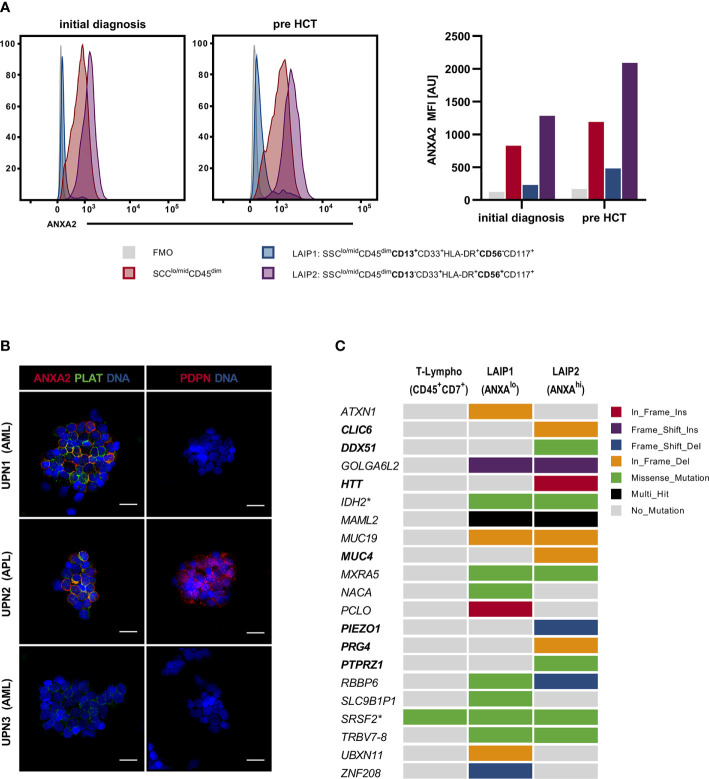
Assessment of patients BM-MNCs *via* MFC, IF and WES. **(A)** Assessment of ANXA2 in UPN1 BM-MNCs populations *via* MFC: BM-MNCs (SSC^lo/mid^CD45^dim^) [red] showed distinct ANXA2 expression on initial presentation as well as pre alloHCT. On closer examination, 2 LAIP were identified: LAIP1 (SSC^lo/mid^CD45^dim^CD13^+^CD33^+^HLA-DR^+^CD56^-^CD117^+^) [blue] showed nearly no ANXA2 expression, whereas LAIP2 (SSC^lo/mid^CD45^dim^ CD13^-^CD33^+^HLA-DR^+^CD56^+^CD117^+^) showed excessive ANXA2 expression [purple]. FMO: negative control [grey]. **(B)** Assessment of ANXA2, PLAT and PDPN *via* IF: BM-MNCs from UPN1 showed marked ANXA2 and PLAT expression, whereas no PDPN expression was observed. BM-MNCs from UPN2 (a patient diagnosed with APL) expressed ANXA2, PLAT as well as PDPN, whereas BM-MNCs from UPN3 (a patient diagnosed with AML (FAB M2) with *KMT2A-*PTD and an *IDH2* mutation without evidence of DIC) neither showed ANXA2 nor PDPN expression, only low-level PLAT expression was observed. Scale bar corresponds to 20µm. **(C)** Mutational profile of LAIP 1 (MFC ANXA2^lo^) and LAIP2 (MFC ANXA2^hi^) as assessed by WES; T-lymphocytes served as a germline-like control. Columns represent LAIPs, rows represent genes and box color indicates the type of genomic alteration. Variants in bold print are exclusively found in LAIP2. Asterisk indicates previously known variants in *IDH2* and *SRSF2*. alloHCT, allogeneic hematopoietic cell transplantation; ANXA2, annexin A2; AU, arbitrary units; BM-MNCs, bone marrow derived mononucleated cells; DIC, disseminated intravascular coagulation; FMO, fluorescence minus-one; LAIP, leukemia-associated immunophenotype; MFC, multiparametric flow cytometry; PLAT, tissue-type plasminogen activator; PDPN, podoplanin; UPN, unique patient number; WES, whole-exome sequencing.

MFC revealed pronounced ANXA2 expression on patients BM-MNCs (SSC^lo/mid^CD45^dim^) (**Figure 2A**). Furthermore, MFC identified two leukemia-associated immunophenotypes (LAIP) with low (LAIP1: SSC^lo/mid^CD45^dim^CD13^+^CD33^+^HLA-DR^+^CD56^-^CD117^+^) and high (LAIP2: SSC^lo/mid^CD45^dim^CD13^-^CD33^+^HLA-DR^+^CD56^+^CD117^+^) ANXA2 expression, respectively (**Figure 2A**). Notably, *via* MFC no ANXA2 expression was seen on healthy donor BM-MNCs (SSC^lo/mid^CD45^dim^) (data not shown).

IF confirmed marked ANXA2 expression on patients BM-MNCs and demonstrated distinct ANXA2/PLAT co-expression, resembling UPN2 (APL) BM-MNCs phenotype (**Figure 2B**). In contrast, PDPN expression was exclusively seen on UPN2 BM-MNCs. BM-MNCs obtained from UPN3 (AML with maturation; evidence of *KMT2A*-PTD and *IDH2* mutation; no evidence of DIC) showed neither ANXA2 nor PDPN expression, arguing against a correlation between evidence of *KMT2A*-PTD and/or *IDH2* mutation and presence of DIC. In contrast, as a possible genotype/phenotype association, DiNardo et al. reported two patients diagnosed with AML with evidence of t(10;11)(p12;q23) translocation (*KMT2A-MLLT10* rearrangement) presenting with DIC (12). However, 1) these patients presented with *KMT2A-MLLT10* fusions, but not *KMT2A*-PTD and 2) in larger studies DIC was not reported as frequent event in adult patients diagnosed with t(v;11q23.3); *KMT2A* rearranged AML (13–15). To further evaluate LAIP2 (MFC ANXA2^hi^) as compared to LAIP1 (MFC ANXA2^lo^) we performed whole exome sequencing (WES) on sorted BM-MNCs populations. Here, T-lymphocytes (SSC^lo^CD45^bright^CD7^+^) served as a germline-like control. WES identified a total 24 variants (4 pathogenic variants, 3 likely pathogenic variants, 17 VUS) in 21 genes including the previously known *IDH2* and *SRSF2* mutations: *ATXN1, CLIC6, DDX51, GOLGA6L2, HTT, IDH2, MAML2, MUC4, MUC19, MXRA5, NACA, PCLO, PIEZO1, PRG4, PTPRZ1, RBBP6, SLC9B1P1, SRSF2, TRBV7-8, UBXN11, ZNF208 -* all variants are reported in detail in **Supplementary Table 1** (**Figure 2C** and **Supplementary Table 1**). Thereof, mutations in the following genes were exclusively seen in LAIP2 (MFC ANXA2^hi^): *CLIC6, DDX51, HTT, MUC4, PIEZO1, PRG4, PTPRZ1.* Here, previous studies found *mastermind like 2 (MAML2)* to be a rare fusion partner of *KMT2A* in patients with myelodysplastic syndromes and secondary AML as well as acute lymphoblastic leukemia (ALL); *mucin 4 (MUC4)* expression to be associated with adverse prognosis in AML and *piezo type mechanosensitive ion channel component 1 (PIEZO1)* frequently mutated in patients with hereditary xerocytosis, hereditary stomatocytosis, respectively (16–18). Although mutational profiling demonstrated high intra-tumor heterogeneity and clonal architecture, genomic characterization could not provide a valid link to the severe coagulopathy with hyperfibrinolysis, coagulation in general, respectively.

Herein we report a non-APL AML patient with clinical and pathophysiological APL features. Although ANXA2 mediated hyperfibrinolysis is a hallmark of APL, this hemorrhagic disorder can also occur in other AML subtypes. In contrast, PDPN expression and associated coagulopathy appears to be specific for APL (7). Therefore, MFC assessment of ANXA2 expression could be a useful tool to determine the risk of potentially life-threatening coagulopathies in patients with newly diagnosed AML. In addition, PDPN should be evaluated as a novel marker of APL/APL-associated hemorrhagic diathesis.

Since complications due to coagulopathies are one of the major causes of morbidity and mortality in AML patients, a high level of vigilance for DIC is indicated (19, 20). According to the “*Management of hemostatic complications in acute leukemia: Guidance from the SSC of the ISTH*” guideline, frequent evaluation for coagulopathy (complete blood count (CBC), PR, aPTT, fibrinogen level, b.i.d.) was performed; further a standardized score was used to diagnose/reevaluate DIC (21). Transfusion support for platelet count <20 x 10^9^/L and fibrinogen level <1,5 g/L was performed. In contrast to the ISTH guideline, which recommends against the routine use of antifibrinolytic agents, tranexamic acid (TA) - a lysine derivate with antifibrinolytic activity by inhibiting the activation of plasminogen - was administered additionally to prevent major hemorrhage. The evidence supporting the use of antifibrinolytic agents in acute leukemia patients remains limited. Despite anecdotal reports claiming beneficial effects of TA in APL patients, the GIMEMA and PETHEMA studies both did not demonstrate differences in incidence of hemorrhagic death as compared to standard treatment (1, 22, 23). However, recent randomized, controlled trials found that administration of TA reduces hemorrhage-associated mortality in post-partum and trauma patients without increasing the risk of thromboembolic events (24, 25). Accordingly, further research regarding usage of TA in the context of AML/APL-associated coagulopathy is warranted.

To conclude, further research to 1) evaluate ANXA2 MFC assessment as a tool to determine the risk of life-threatening DIC in AML patients, 2) investigate PDPN as a marker of APL/APL-associated coagulopathy, 3) determine the efficacy and safety of antifibrinolytic agents in preventing hemorrhage in people with hematological malignancies, and 4) understand the genetic background and upstream regulation of ANXA2 and PLAT and coagulopathy-initiation in non-APL AML and APL clones, is indicated.

## Data Availability Statement

The datasets presented in this article are not readily available because ethics restrictions apply. Requests to access the datasets should be directed to the corresponding author.

## Ethics Statement

The studies involving human participants were reviewed and approved by TU Dresden ethics committee (EK98032010). The patients/participants provided their written informed consent to participate in this study.

## Author Contributions

LR, FS, and MB conceived the presented idea. LR, FS, LW, and MvB performed research. HA provided BM-MNC. AR and ES helped to perform molecular analysis. SH helped to assess WES data. LR wrote the manuscript with help from FS, UP, SH, JS, MB, and MvB. All authors contributed to the article and approved the submitted version.

## Conflict of Interest

The authors declare that the research was conducted in the absence of any commercial or financial relationships that could be construed as a potential conflict of interest.

## References

[1] de la Serna J Montesinos P Vellenga E Rayón C Parody R León A. Causes and prognostic factors of remission induction failure in patients with acute promyelocytic leukemia treated with all-trans retinoic acid and idarubicin. Blood (2008) 111(7):3395–402. 10.1182/blood-2007-07-100669 18195095

[2] Tallman MS Kwaan HC. Reassessing the hemostatic disorder associated with acute promyelocytic leukemia. Blood (1992) 79(3):543–53. 10.1182/blood.V79.3.543.543 1732003

[3] Menell JS Cesarman GM Jacovina AT McLaughlin MA Lev EA Hajjar KA. Annexin II and Bleeding in Acute Promyelocytic Leukemia. N Engl J Med (1999) 340(13):994–1004. 10.1056/NEJM199904013401303 10099141

[4] Hajjar KA Jacovina AT Chacko J. An endothelial cell receptor for plasminogen/tissue plasminogen activator. I. Identity with annexin II. J Biol Chem (1994) 269(33):21191–7. 10.1016/S0021-9258(17)31947-6 8063740

[5] Liu Y Wang Z Jiang M Dai L Zhang W Wu D. The expression of annexin II and its role in the fibrinolytic activity in acute promyelocytic leukemia. Leuk Res (2011) 35(7):879–84. 10.1016/j.leukres.2010.11.008 21146216

[6] Breiteneder-Geleff S Soleiman A Kowalski H Horvat R Amann G Kriehuber E. Angiosarcomas express mixed endothelial phenotypes of blood and lymphatic capillaries: podoplanin as a specific marker for lymphatic endothelium. Am J Pathol (1999) 154(2):385–94. 10.1016/S0002-9440(10)65285-6 PMC1849992 10027397

[7] Lavallée V-P Chagraoui J MacRae T Marquis M Bonnefoy A Krosl J. Transcriptomic landscape of acute promyelocytic leukemia reveals aberrant surface expression of the platelet aggregation agonist Podoplanin. Leukemia (2018) 32(6):1349–57. 10.1038/s41375-018-0069-1 29550835

[8] Toh CH Hoots WK. SSC on Disseminated Intravascular Coagulation of the ISTH. The scoring system of the Scientific and Standardisation Committee on Disseminated Intravascular Coagulation of the International Society on Thrombosis and Haemostasis: a 5-year overview. J Thromb Haemost JTH (2007) 5(3):604–6. 10.1111/j.1538-7836.2007.02313.x 17096704

[9] Grimwade D Ivey A Huntly BJP. Molecular landscape of acute myeloid leukemia in younger adults and its clinical relevance. Blood (2016) 127(1):29–41. 10.1182/blood-2015-07-604496 26660431 PMC4705608

[10] Kopanos C Tsiolkas V Kouris A Chapple CE Albarca Aguilera M Meyer R. VarSome: the human genomic variant search engine. Bioinforma Oxf Engl (2019) 35(11):1978–80. 10.1093/bioinformatics/bty897 PMC6546127 30376034

[11] Richards S Aziz N Bale S Bick D Das S Gastier-Foster J. Standards and guidelines for the interpretation of sequence variants: a joint consensus recommendation of the American College of Medical Genetics and Genomics and the Association for Molecular Pathology. Genet Med Off J Am Coll Med Genet (2015) 17(5):405–24. 10.1038/gim.2015.30 PMC4544753 25741868

[12] DiNardo CD Tang G Pemmaraju N Wang SA Pike A Garcia-Manero G. Acute myeloid leukemia with t(10;11): a pathological entity with distinct clinical presentation. Clin Lymphoma Myeloma Leuk (2015) 15(1):47–51. 10.1016/j.clml.2014.06.022 25081372 PMC4849878

[13] Chen Y Kantarjian H Pierce S Faderl S O’Brien S Qiao W. Prognostic significance of 11q23 aberrations in adult acute myeloid leukemia and the role of allogeneic stem cell transplantation. Leukemia (2013) 27(4):836–42. 10.1038/leu.2012.319 PMC4181539 23135353

[14] Bill M Mrózek K Kohlschmidt J Eisfeld A-K Walker CJ Nicolet D. Mutational landscape and clinical outcome of patients with *de novo* acute myeloid leukemia and rearrangements involving 11q23/KMT2A. Proc Natl Acad Sci (2020) 117(42):26340–6. 10.1073/pnas.2014732117 PMC7584992 33020282

[15] Hinai ASAA Pratcorona M Grob T Kavelaars FG Bussaglia E Sanders MA. The Landscape of KMT2A-PTD AML: Concurrent Mutations, Gene Expression Signatures, and Clinical Outcome. HemaSphere (2019) 3(2):e181. 10.1097/HS9.0000000000000181 31723820 PMC6746036

[16] Nemoto N Suzukawa K Shimizu S Shinagawa A Takei N Taki T. Identification of a novel fusion gene MLL-MAML2 in secondary acute myelogenous leukemia and myelodysplastic syndrome with inv(11)(q21q23). Genes Chromosomes Cancer (2007) 46(9):813–9. 10.1002/gcc.20467 17551948

[17] Abdelhady AS Abdel Hamid FF Hassan NM Ibrahim DM. Prognostic value of bone marrow MUC4 expression in acute myeloid leukaemia. Br J BioMed Sci (2020) 77(4):202–7. 10.1080/09674845.2020.1754583 32270747

[18] Zarychanski R Schulz VP Houston BL Maksimova Y Houston DS Smith B. Mutations in the mechanotransduction protein PIEZO1 are associated with hereditary xerocytosis. Blood (2012) 120(9):1908–15. 10.1182/blood-2012-04-422253 PMC3448561 22529292

[19] Törnebohm E Lockner D Paul C. A retrospective analysis of bleeding complications in 438 patients with acute leukaemia during the years 1972–1991. Eur J Haematol (1993) 50(3):160–7. 10.1111/j.1600-0609.1993.tb00085.x 8472811

[20] Yanada M Matsushita T Suzuki M Kiyoi H Yamamoto K Kinoshita T. Disseminated intravascular coagulation in acute leukemia: clinical and laboratory features at presentation. Eur J Haematol (2006) 77(4):282–7. 10.1111/j.1600-0609.2006.00711.x 16856920

[21] Wang T-F Makar RS Antic D Levy JH Douketis JD Connors JM. Management of hemostatic complications in acute leukemia: Guidance from the SSC of the ISTH. J Thromb Haemost JTH (2020) 18(12):3174–83. 10.1111/jth.15074 PMC7909744 33433069

[22] Rodeghiero F Avvisati G Castaman G Barbui T Mandelli F. Early deaths and anti-hemorrhagic treatments in acute promyelocytic leukemia. A GIMEMA retrospective study in 268 consecutive patients. Blood (1990) 75(11):2112–7. 10.1182/blood.V75.11.2112.2112 2189506

[23] Sugawara T Okuda M Yamaguchi Y Endo K Yoshinaga K. Successful treatment with tranexamic acid for severe bleeding in acute promyelocytic leukemia. Acta Haematol (1992) 87(1–2):109. 10.1159/000204732 1585765

[24] WOMAN Trial Collaborators. Effect of early tranexamic acid administration on mortality, hysterectomy, and other morbidities in women with post-partum haemorrhage (WOMAN): an international, randomised, double-blind, placebo-controlled trial. Lancet Lond Engl (2017) 389(10084):2105–16. 10.1016/S0140-6736(17)30638-4 PMC5446563 28456509

[25] CRASH-2 trial collaborators Shakur H Roberts I Bautista R Caballero J Coats T. Effects of tranexamic acid on death, vascular occlusive events, and blood transfusion in trauma patients with significant haemorrhage (CRASH-2): a randomised, placebo-controlled trial. Lancet Lond Engl (2010) 376(9734):23–32. 10.1016/S0140-6736(10)60835-5 20554319

