# Supplementary material for: Case Report: ANXA2 Associated Life-Threatening Coagulopathy With Hyperfibrinolysis in a Patient With Non-APL Acute Myeloid Leukemia
Source: Front Oncol. 2021 Apr 15;11:666014. doi: 10.3389/fonc.2021.666014 (PMC8082174; doi:10.3389/fonc.2021.666014)
Supplement: Supplementary file 1 [file Table_1.docx]

| **Supplementary Table 1** | | | | | |
| --- | --- | --- | --- | --- | --- |
| **Gene (HGNC)** | **Variant**  **(HGVS)** | **Cytogenetic location (ISCN)** | **refSNP ID**  **(dbSNP)** | **Interpretation (ACMG)** | **Literature** |
| ***ATXN1*** | ATXN1(ENST00000436367.1):c.627_635delTCAGCATCA (p.His209_His211del) | 6p22.3 | rs776272024 | VUS |  |
| ***CLIC6*** | CLIC6(ENST00000360731.3):c.775_804del  (p.Gly259_Asp268del) | 21q22.12 | rs1317456482 | VUS |  |
| ***DDX51*** | DDX51(ENST00000397333.3):c.347G>A  (p.Ser116Asn) | 12q24.33 | rs1194651659 | VUS | PMID: 17363581 |
| ***GOLGA6L2*** | GOLGA6L2(ENST00000312015.5):c.1632+67_1632+68insAAGCAG  GAGGAGAAGATGCG | 15q11.2 |  | VUS |  |
| ***HTT*** | HTT(ENST00000355072.5):c.120_125dupACCGCC  (p.Pro48_Pro49dup) | 4p16.3 |  | VUS |  |
| ***IDH2*** | IDH2(ENST00000330062.3):c.419G>A  (p.Arg140Gln) | 15q26.1 | rs121913502 | pathogenic | PMID: 30967620; PMID: 28588020 |
| ***MAML2*** | MAML2(ENST00000524717.1):c.1907_1914delTTTCAGCC (p.Ile636ThrfsTer92) | 11q21 |  | likely pathogenic | PMID: 17551948 |
| ***MAML2*** | MAML2(ENST00000524717.1):c.1900_1903delAGCT  (p.Ser634GlnfsTer46) | 11q21 |  | likely pathogenic | PMID: 17551948 |
| ***MAML2*** | MAML2(ENST00000524717.1):c.1918C>T  (p.Gln640Ter) | 11q21 |  | pathogenic | PMID: 17551948 |
| ***MUC4*** | MUC4(ENST00000349607.4):c.83-8111_83-8016del | 3q29 |  | VUS | PMID: 32270747 |
| ***MUC19*** | MUC19(ENST00000454784.4):c.8461_8490del  (p.Arg2821_Thr2830del) | 12q12 |  | VUS |  |
| ***MXRA5*** | MXRA5(ENST00000217939.6):c.7309G>A  (p.Val2437Ile) | Xp22.33 | rs41309655 | VUS |  |
| ***NACA*** | NACA(ENST00000356769.3):c.71-3762_71-3760delATTinsCCC | 12q13.3 |  | VUS |  |
| ***PCLO*** | PCLO(ENST00000333891.9):c.1125_1126insCCTCCAGCTCAGCAC (p.Gln375_Thr376insProProAlaGlnHis) | 7q21.11 |  | VUS |  |
| ***PIEZO1*** | PIEZO1(ENST00000301015.9):c.1297-50_1298del | 16q24.3 |  | pathogenic | PMID: 22529292 |
| ***PRG4*** | PRG4(ENST00000367482.4):c.1014_1037del  (p.Thr344_Thr351del) | 1q31.1 |  | VUS |  |
| ***PTPRZ1*** | PTPRZ1(ENST00000449182.1):c.2263+1440A>T  (p.Met1235Leu) | 7q31.32 |  | VUS |  |
| ***RBBP6*** | RBBP6(ENST00000319715.4):c.3255delT  (p.Gly1086GlufsTer25) | 16p12.1 |  | likely pathogenic | PMID: 26574608 |
| ***RBBP6*** | RBBP6(ENST00000319715.4):c.2856G>T  (p.Glu952Asp) | 16p12.1 |  | VUS | PMID: 26574608 |
| ***SLC9B1P1*** | SLC9B1P1(ENST00000331172.6):c.400T>C  (p.Phe134Leu) | Yq11.21 | rs774937896 | VUS |  |
| ***SRSF2*** | SRSF2(ENST00000359995.5):c.284C>A  (p.Pro95His) | 17q25.1 | rs751713049 | pathogenic | PMID: 32358566 |
| ***TRBV7-8*** | TRBV7-8(ENST00000390359.3):c.213_215delAGCinsCAG (p.Glu71_Ala72delinsAspSer) | 7q34 |  | VUS |  |
| ***UBXN11*** | UBXN11(ENST00000314675.7):c.1104_1181del  (p.Gly370_Pro395del) | 1p36.11 |  | VUS |  |
| ***ZNF208*** | ZNF208(ENST00000599916.1):c.305+1delG | 19p12 |  | VUS |  |
